# The Effect of Ozone on the Behavior of Systemic and Non-Systemic Pesticides in Cereal Grains

**DOI:** 10.3390/molecules30204087

**Published:** 2025-10-14

**Authors:** Izabela Hrynko

**Affiliations:** Laboratory of Food and Feed Safety, Institute of Plant Protection-National Research Institute, Chelmonskiego 22 St., 15-195 Bialystok, Poland; i.hrynko@iorpib.poznan.pl

**Keywords:** systemic and non-systemic pesticides, ozone, cereal grains, processing factor

## Abstract

Cereal grains make up a significant part of both human and animal diets; therefore, they should meet pesticide residue standards and be characterized by the lowest possible concentrations of these residues. Known for its strong oxidizing properties, ozone is gaining popularity as a natural agent for eliminating chemical contaminants at the stages of production, processing, and storage of raw materials of plant origin. The present study is the first to assess the effect of ozonation on the behavior of 12 (seven systemic and five non-systemic) compounds. The procedure was conducted in two time variants (30 and 60 min) for three cereal types: barley, wheat, and rye. Treatment efficiency was confirmed through instrumental determination conducted using the LC–MS/MS technique based on the QuEChERS protocol. The level of systemic compounds was reduced by 37–82%, and of non-systemic ones by approximately 72–95%. The reduction in difenoconazole amounted to only 39%, whereas the highest decrease of 95% was recorded for deltamethrin. The rate of pesticide degradation occurred in the following sequence: rye > wheat > barley. The results show that ozonation of cereal grains may successfully support assurance of food and feed safety.

## 1. Introduction

Recent years have seen a systematic increase in the number of pests causing significant losses in cereal crops. The most commonly encountered diseases include powdery mildew (*Erysiphe graminis*), brown rust (*Puccinia recondita*), stripe rust (*Puccinia striiformis*), fusarium ear blight (*Fusarium* spp.), septoria leaf spots (*Septoria* spp.), and pink snow mold (*Microdochium nivale*) [1,2]. The major pests of cereal crops include grain aphids (*Aphidomorpha*), snout beetles (*Curculionidae*), cutworms (*Agrotis* spp.), and cabbage-stem flea beetles (*Psylliodes chrysocephala*) [3,4,5]. In the face of such threats, farmers reach for plant protection chemicals. However, these are increasingly used in excess amounts, in violation of the safety rules and guidelines in force [6].

Due to their toxicity, pesticides pose a threat to both the natural environment and human health [7,8]. There is increasing evidence of a relationship between exposure to pesticides and incidence of chronic diseases in humans, including cancer, Parkinson’s and Alzheimer’s diseases, multiple sclerosis, diabetes, and cardiovascular diseases [9,10,11]. Responding to the growing concerns related to the presence of pesticide residues in agricultural products and the risk of effect thereof on humans, as well as to the growing interest in non-chemical plant protection methods, researchers in many countries have intensified their works on utilization of ozone [12,13,14]. With its strong oxidizing properties, ozone (O_3_) finds application as a natural agent for pesticide residue reduction at the stages of production, processing, and storage stability extension of cereals and raw materials of plant origin [15,16,17]. Since the degradation product of ozone is oxygen, application thereof causes no secondary environmental pollution [18].

Ozonation can be used commercially by cereal processors, farmers, and seed producers as a non-chemical method of protecting stored grain. Thanks to its flexibility, ozonation has the potential to bring significant savings in operating costs [19]. This method is already being used in some countries to protect stored grain in an environmentally safe manner [20].

In practice, two main approaches are employed: washing or immersion of raw materials of plant origin in ozone-enriched water, or subjecting the products to the effect of ozone in gaseous form—either continuously or periodically [21]. Both forms differ in their mechanism of action, as ozone exhibits distinct properties in water and in air and reacts differently to environmental factors. The efficiency of ozonation is affected by pH, temperature, humidity, ozone concentration, its form of application, and the chemical characteristics of the given compounds [22]. Aqueous ozonation, with the simultaneous effect of ozone and hydroxyl radicals occurring in water, usually results in quicker and more efficient degradation of contaminants. Gaseous ozonation, on the other hand, typically requires longer exposure or higher concentrations but has the advantage of reaching hard-to-access areas, making it particularly useful for the storage and disinfection of raw materials [23].

The susceptibility of food to ozone varies depending on its type and species within a given type. Ozone penetrates fruit more easily because it has high water content and a soft, permeable skin. Cereals have a hard, dry, and compact coating that can act as a barrier to ozone penetration [24]. Many researchers have reported that ozonation is effective in removing pesticide residues, and this has been confirmed for agricultural products such as citrus [25], pepper [26], lettuce [27,28], tomatoes [29,30], apples [31,32], carrots [18], grapes [33,34,35], strawberries [36], blackcurrant [37], and maize [38]. However, most of these studies focus on aqueous ozonation, while only a few address the application of ozone in gaseous form. Gabler et al. [34] proved the high efficiency of ozonation of grapes using ozone in gaseous form. After 60 min of exposure to ozone with a concentration of 10 mL L^−1^, significant degradation of pesticide residues was observed; in the case of pyraclostrobin, cyprodinil, fenhexamid, and pyrimethanil, it was between 68.5% and 100%. According to Amjad et al. [35], the use of ozone enabled the removal of two pesticides (chlorpyriphos and imidacloprid) from the surface of grapes in the range of 75–95%. Similarly, Heleno et al. [33] confirmed that using two different ozone concentrations (3 mg L^−1^ and 2 mg L^−1^) reduced pesticide residues in grapes by approximately 60%.

Due to the widespread consumption of cereal products and long-term storage of grain, the presence and potential accumulation of pesticide residues pose a significant hazard to food safety [39]. As a result, the assessment of the usefulness of ozonation as a safe and efficient method for reducing pesticide residues in cereals is of special importance to both consumer protection and safety assurance of cereal products. Although the potential of ozone in the removal of pesticide residues has been confirmed for many food products, there are still not enough reports on the removal of pesticide residues from cereals. In relation to cereals, the scope of such analyses remains limited. The objective of the present study was to assess the effect of ozone on the behavior of twelve selected systemic and non-systemic pesticides in grains of three cereal species. This is the first study conducted on such a broad scale concerning analysis of the effect of ozonation on cereals.

## 2. Results

### 2.1. Method Validation Results

Recovery ranges, average recovery ranges, and their corresponding relative standard deviation (RSD), obtained for 12 pesticides, are shown in Table 1. As evident from the data, recoveries ranged from 70% to 118%, with the standard deviation calculated for five replicates being less than 16% in all cases. The matrix effect values obtained ranged from −10% to 17%. The expanded measurement uncertainty was 4% to 18%.

### 2.2. The Effect of Ozone on the Behavior of Pesticides in Cereal Grains

Immediately after the “pesticide bath,” the residues of pesticides before ozonation were determined. The results indicated levels safe for human consumption and are presented in Table 2. The estimated initial concentrations of the 12 pesticides fell within the range between 0.1864 mg kg^−1^ (deltamethrin/barley) and 0.9656 mg kg^−1^ (cypermethrin/wheat).

**Figure 1:**
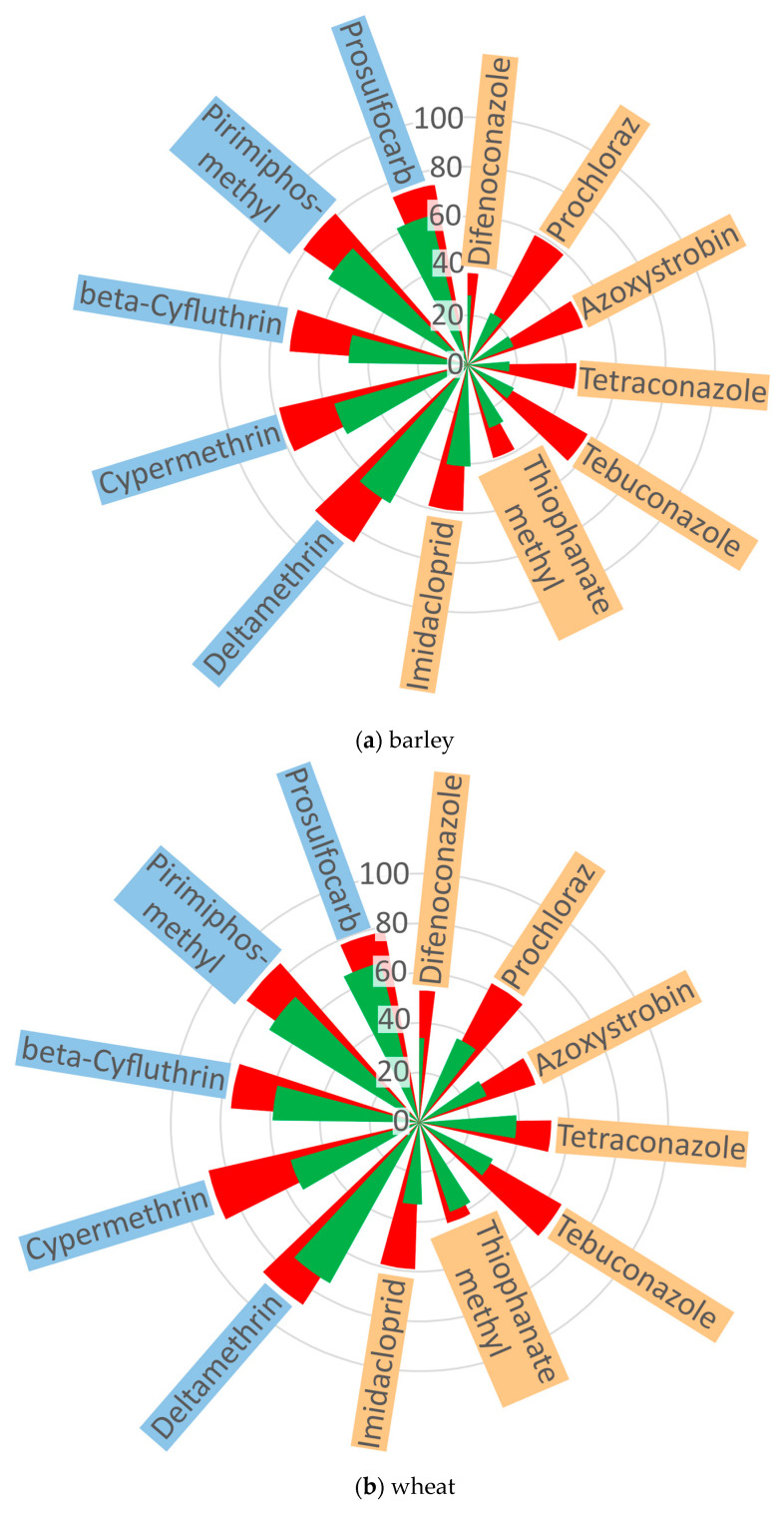

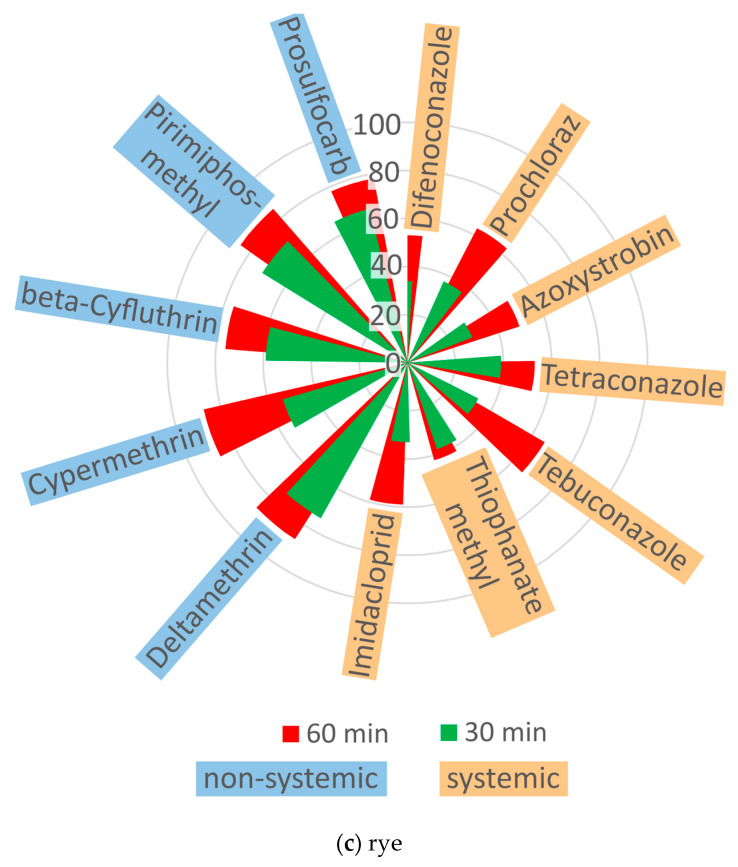
Reduction in residues of systemic and non-systemic pesticides in cereals by ozonation (%) (n = 3).

The presence of all the pesticides under analysis was determined in the cereals, and the content thereof gradually decreased after 30 and 60 min of ozonation. Each pesticide showed a steady decline in content (Table 2). The largest residue reduction was obtained for deltamethrin, a non-systemic insecticide (a compound from group VII) in rye cereal, following ozonation for 60 min. In contrast, the lowest reduction was found for tetraconazole (a compound from group III) in barley following 5 min ozonation. The effect of ozonation on the residues of all 12 pesticides for three cereal species is presented in Table 2 and Figure 1.

It can be assumed that the efficiency of ozonation may have been influenced by the systemic properties of the compounds, reflecting their potential for translocation within cereal grains. Removal of non-systemic pesticides (deltamethrin, beta-cyfluthrin, cypermethrin, pirimiphos-methyl, and prosulfocarb) through ozonation was easier in comparison with difenoconazole, azoxystrobin, tetraconazole, tebuconazole, thiophanate-methyl, imidacloprid, and prochloraz, all of which exhibit a systemic effect.

After 30 min of ozonation, over 50% reduction for systemic insecticides was only recorded for prochloraz (51%) and difenoconazole (52%) in rye grain. In the case of non-systemic insecticides, all were reduced by more than 50%, except beta-cyfluthrin in barley, for which the reduction was 48%.

After 60 min of ozonation, further degradation of the pesticides fell within the range of 12–35%. The content of systemic imidacloprid and tebuconazole decreased, on average, to 63% of the content prior to ozonation. High reductions, exceeding 70%, were recorded for non-systemic insecticides: deltamethrin (89%), beta-cyfluthrin (75%), cypermethrin (83%), pirimiphos-methyl (87%), and prochloraz (78%) (Figure 1).

Analysis of chemical groups of pesticides showed that the residue levels of those from group III (triazoles), such as tetraconazole and tebuconazole, were reduced after 60 min ozonation by 44%, 53%, and 59% for tetraconazole, as well as by 56%, 66%, and 68% for tebuconazole in barley, wheat, and rye grain, respectively. For comparison, the compounds of group VI (pyrethroids)—deltamethrin, beta-cyfluthrin, and cypermethrin—showed significantly higher reductions after an identical ozonation time: 85%, 87%, and 95% for deltamethrin; 72%, 76%, and 77% for beta-cyfluthrin; and 78%, 87%, and 85% for cypermethrin (Figure 1).

Since the tests were conducted on grains of three different cereal species characterized by different seed coat structures, it was observed that the cereals were ranked as follows in terms of pesticide degradation: rye > wheat > barley. Barley showed reductions of between 17% (tetraconazole/30 min) and 85% (deltamethrin/60 min). Barley has a thicker husk and a more compact structure, which may slow down the degradation of systemic compounds such as tetraconazole. In wheat, the level of pesticides was reduced by between 29% (azoxystrobin/30 min) and 87% (deltamethrin/cypermethrin 60 min). In rye grain, the pesticide content was reduced by between 29% (tebuconazole/30 min) and 95% (deltamethrin/60 min). Rye has a thinner coat and a more porous structure, which may be conducive to quicker degradation of pesticides present mainly on the grain’s surface, which are more susceptible to the oxidizing effect of ozone. Since the grain moisture content was maintained below 14% for all cereal types, differences in moisture could not have influenced the degradation of the compounds.

### 2.3. Processing Factors

**Figure 2:**
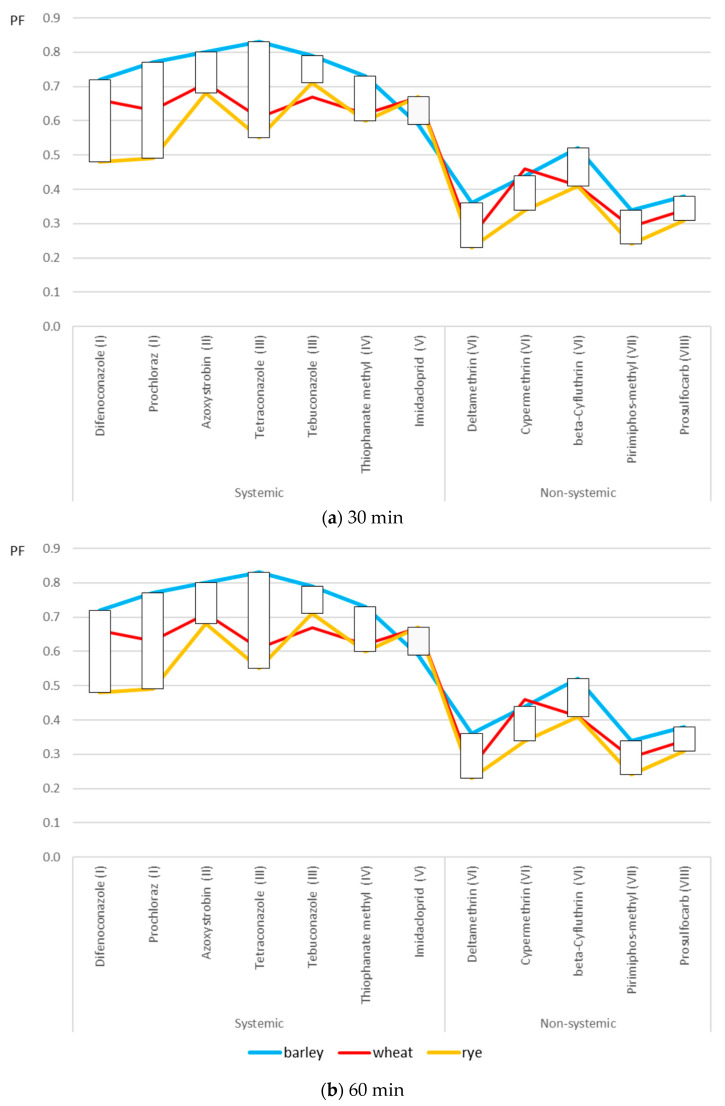
Overall processing factors (PFs) for the ozonation of three cereal types at two time variants ((**a**): 30 min; (**b**): 60 min).

Figure 2 shows the calculated processing factors (PFs) for 12 pesticides during an ozonation process performed in two time variants (30 and 60 min). Generally speaking, the PF values for both ozonation variants were lower than 1, suggesting that reduction in pesticide levels was achieved during these processes.

In the case of 30 min ozonation, PF values were between 0.48 and 0.83 for systemic pesticides and between 0.23 and 0.52 for non-systemic pesticides, which suggests only a slight reduction in their residues.

In contrast, for 60 min ozonation, the observed PF values fluctuated between 0.18 and 0.61 for systemic pesticides, as well as between 0.05 and 0.28 for non-systemic pesticides.

In the case of deltamethrin and pirimiphos-methyl (with PFs of 0.05 and 0.06, respectively), ozonation significantly reduced the residue levels.

For both 30 min and 60 min ozonation, PF values, depending on the cereal type, showed broader distribution for certain compounds, e.g., between 0.49 and 0.77 (prochloraz), between 0.55 and 0.83 (tetraconazole) for shorter ozonation, between 0.39 and 0.63 (difenoconazole), and between 0.18 and 0.51 (imidacloprid) for longer ozonation.

## 3. Discussion

In the present study, the highest initial concentration was found for cypermethrin, the level of which fell by 78–87% following 60 min ozonation. This is likely due to the fact that ozone, as an oxidizing agent, decomposes the structure of cypermethrin through a reaction with the double bonds present in its molecule [40]. An additional factor contributing to the efficiency of this process is that cypermethrin is a non-systemic insecticide, having a local effect at the point of contact with the plant surface, with no ability to penetrate inside [41]. Therefore, residues of this substance mainly accumulate on the surface of grains, making them more accessible to ozone and thus more susceptible to its effects. In the case of deltamethrin, another compound from the pyrethroid group (group VI; Table 3), a high reduction level was obtained as well, falling within the range of 85–95%. Similar observations for compounds from group VI were observed by Savi et al. [42], who studied the effect of ozonation on the removal of deltamethrin from wheat grains. They demonstrated that the deltamethrin content was reduced by 90%, albeit only after approximately 3 h of exposure to ozone in gaseous form. De Ávila et al. [43] also confirmed the high efficiency of ozonation in gaseous form in removing deltamethrin and bifenthrin from rice grains, achieving reductions of up to 90%.

Another insecticide commonly used in field crops and in the protection of stored grain, pirimiphos-methyl [44], had its concentration reduced by 81–94% after a 60 min ozonation. This pesticide, representing organophosphorus compounds (group VII; Table 3), is characterized by high lipophilicity, causing it to mainly accumulate on grain surfaces [45]. Such a location makes pirimiphos-methyl, similarly to another organophosphorus compound, chlorpyriphos, particularly exposed to the direct effect of ozone, which efficiently degrades organic substances found on the surface. This finding is consistent with previous studies, as the ozonation process was found to contribute significantly to the reduction in pirimiphos-methyl residues in maize grains, lowering their level by as much as 91% [38]. The efficiency of this insecticide’s degradation was found to increase proportionally to the time of exposure to ozone. Other researchers recorded variable degradation efficiency for compounds from group VII as a result of ozonation of wheat grains [46], chili peppers [47], citruses [25], and strawberries [36], falling within the range of 51–94%.

In contrast, systemic pesticides, such as difenoconazole and prochloraz (group I; Table 3), azoxystrobin (group II), tetraconazole and tebuconazole (group III), or imidacloprid (group V), were harder to remove. In the case of grain cereals, this means the active substances of these pesticides may not only be found on the surface but also penetrate into deeper layers. Since ozone has a largely superficial effect, its efficiency in degrading systemic pesticides is significantly lower compared to non-systemic compounds [26]. Triazoles (group III; Table 3) are characterized by the presence of stable heterocyclic rings and are more resistant to the effect of ozone [48]. Their chemical structure is less reactive to ozone, resulting in slower particle decomposition [49]. Tetraconazole is a compound with a higher molecular mass (M = 372.15 g mol^−1^) than tebuconazole (M ≤ 307.82 g mol^−1^); therefore ozonation was slightly less efficient in the removal of this pesticide (PF = 0.41–0.56 for tetraconazole and PF = 0.32–0.44 for tebuconazole; Table 3). A higher molecular mass may be conducive to higher chemical stability and lower reactivity with ozone [50].

Compounds representing conazoles (group I; Table 3), difenoconazole, and prochloraz, had their concentrations reduced by 37%, 53%, and 61% (difenoconazole) as well as by 59%, 63% and 65% (prochloraz) in barley, wheat, and rye grain, respectively. A slightly higher reduction in concentration for difenoconazole (95%) was observed by Heleno et al. [33], who tested the effect of ozonation on the removal of this fungicide from strawberries. In this case, due to their soft surface and high water content, these berries could be conducive to the decomposition of the fungicide. A similar situation was described by Rodrigues et al. [51], where ozonation of tomatoes resulted in a reduction in residues of azoxystrobin, chlorothalonil, and difenoconazole within the range of 70–90%.

It is assumed that prolonging exposure to ozone or using higher concentrations of it may further increase the rate of pesticide decomposition. A study on permethrin (a pyrethroid insecticide) and chlorothalonil (a chloroorganic fungicide) used on Chinese cabbage showed that the rate of degradation of these compounds increased with increasing ozone concentration [52]. Similar relationships were observed in the case of difenoconazole (a triazole fungicide) and linuron (a urea herbicide) used on carrots [18]. Other researchers have also noted a positive correlation between the effectiveness of pesticide residue degradation and the duration of ozonation. Savi et al. [42] showed that in the case of wheat grain ozonation, extending the exposure time from 30 to 180 min resulted in a slight but noticeable increase in the rate of deltamethrin decomposition. However, it is worth noting that excessive exposure of food to ozone may negatively affect its quality properties, as pointed out by Wang’s study [23].

**Table 3:** Structures and physicochemical properties of the 12 pesticides under investigation.

Pesticide	Group	Chemical Group	Formula	Structure	M (g mol^−1^)	log P
systemic						
Difenoconazole	I	Conazole	C_19_H_17_Cl_2_N_3_O_3_	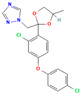	406.26	4.36
Prochloraz	I	Conazole	C_15_H_16_Cl_3_N_3_O_2_	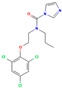	376.7	3.5
Azoxystrobin	II	Strobilurin	C_22_H_17_N_3_O_5_	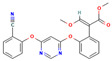	403.4	2.5
Tetraconazole	III	Triazole	C_13_H_11_Cl_2_F_4_N_3_O	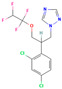	372.15	3.56
Tebuconazole	III	Triazole	C_16_H_22_ClN_3_O	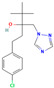	307.82	3.7
Thiophanate methyl	IV	Carbamate	C_12_H_14_N_4_O_4_S_2_	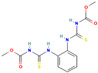	342.39	1.4
Imidacloprid	V	Neonicotinoid	C_9_H_10_ClN_5_O_2_	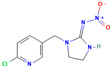	255.66	0.57
non-systemic						
Deltamethrin	VI	Pyrethroid	C_22_H_19_Br_2_NO_3_	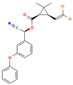	505.2	4.6
Cypermethrin	VI	Pyrethroid	C_22_H_19_Cl_2_NO_3_	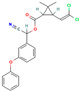	416.3	5.55
beta-Cyfluthrin	VI	Pyrethroid	C_22_H_18_Cl_2_FNO_3_	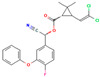	416.3	5.8
Pirimiphos-methyl	VII	Organophosphate	C_11_H_20_N_3_O_3_PS	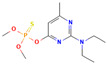	305.33	4.2
Prosulfocarb	VIII	Thiocarbamate	C_14_H_21_NOS	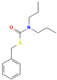	251.39	4.48

M: molar mass; log P: logarithm of the octanol-water partition coefficient (pH = 7, temperature at 20 °C); Source: PPDB, 2025 [53].

## 4. Materials and Methods

### 4.1. Chemicals

Twelve analytical standards, difenoconazole (CAS number 119446-68-3), prochloraz (67747-09-5), azoxystrobin (131860-33-8), tetraconazole (112281-77-3), tebuconazole (107534-96-3), thiophanate methyl (23564-05-8), imidacloprid (138261-41-3), deltamethrin (52918-63-5), cypermethrin (52315-07-8), beta-cyfluthrin (1820573-27-0), pirimiphos-methyl (29232-93-7), and prosulfocarb (52888-80-9) with up to 95% purity (Table 3), were purchased from Dr. Ehrenstorfer GmbH (Augsburg, Germany). Triphenyl phosphate (TPP) and isoproturon-d6, as internal standards (ISs), were supplied by Sigma-Aldrich (St. Louis, MO, USA).

Stock standard solutions of pesticides were prepared separately by dissolving an accurately weighed amount of each reference standard in acetone at a concentration of approximately 1000 mg mL^−1^. The combined working standard solutions were generated by serial dilution of the stock solutions with methanol. TPP and isoproturon-d6 solutions were prepared as described above. All stock and working standard solutions were protected from direct light and stored in dark glass bottles in a freezer at about −4 °C until analysis.

Acetonitrile, methanol, and formic acid were of LC–MS grade and purchased from Merck (Darmstadt, Germany). Ultrapure water was derived from a Milli-Q ultrapure water purification system (Millipore, Darmstadt, Germany). The QuEChERS extraction packet was sourced from Agilent Technologies (Santa Clara, CA, USA). Dispersive solid-phase extraction (d-SPE) sorbents containing MgSO_4_, PSA, and C18 were sourced from Agilent Technologies (Santa Clara, CA, USA).

### 4.2. Spiking of Cereal Grain Samples

Grain samples of wheat, rye, and barley (10 kg each) were immersed separately for 4 h in solutions of individual plant protection chemicals. The pesticides were diluted in 5 L of water in order to prepare a working solution—a “pesticide bath”—in line with the manufacturer’s recommendations. The experiments conducted are of a model nature. The concentration and exposure time were selected in such a way as to achieve the desired residue levels of 12 pesticides without adversely affecting the environment. Furthermore, the process itself was designed so that the results obtained reflect those obtained in real-life conditions as closely as possible.

The active substances and their doses in 5 L of water included: difenoconazole (250 g L^−1^, 3 mL), prochloraz (450 g L^−1^, 5 mL), azoxystrobin (250 g L^−1^, 5 mL), tetraconazole (100 g L^−1^, 0.6 mL), tebuconazole (250 g L^−1^, 1.5 mL), thiophanate methyl (500 g L^−1^, 1.7 mL), imidacloprid (200 g L^−1^, 0.8 mL), deltamethrin (50 g L^−1^, 0.4 mL), cypermethrin (500 g L^−1^, 0.3 mL), beta-cyfluthrin (25 g L^−1^, 1.7 mL), pirimiphos-methyl (500 g L^−1^, 11 mL), and prosulfocarb (800 g L^−1^, 15 mL). Upon completion of the immersion, the grain was drained of the solution of each plant protection chemical and rinsed three times with distilled water.

### 4.3. Ozonation

Layers of wheat, barley, and rye grains, each 1 cm thick, were placed on a flat table in a tightly sealed chamber under controlled conditions, with temperature maintained at 15 ± 1 °C and relative humidity maintained at 65 ± 5% and monitored in real time. Grain ozonation was carried out in a sealed chamber, which allowed for monitoring of key parameters such as ozone concentration, temperature, and humidity. This ensured uniform and repeatable conditions for all tests. The influence of external factors such as air flow and temperature fluctuations was also reduced. The ozone concentration in the chamber was monitored using an ozone meter (OzonMED, Gorzów Wielkopolski, Poland), which measured ozone concentration in real time. The moisture content in the grain, both before and after treatment, was determined using the weighing-drying method and maintained below 14%.

GL-2186 (Shenzhen Guanglei Electronic, Shenzhen, China), with a production capacity of 20 g h^−1^, was used for ozonation. Ozone was generated and introduced into the 15.75 m^3^ chamber via a polyvinyl chloride (PVC) tube with an inner diameter of 10 mm. The air/ozone flow rate through the chamber was maintained at 20 L min^−1^ with an inner diameter of 10 mm. The air/ozone flow rate through the chamber was maintained at 20 L min^−1^. To ensure representative sampling, grains were collected from ten different locations and combined to form a composite sample for subsequent analyses. The experiments were conducted for two different ozonation durations, and each treatment was repeated three times for each cereal type.

### 4.4. Sample Preparation

**Figure 3:**
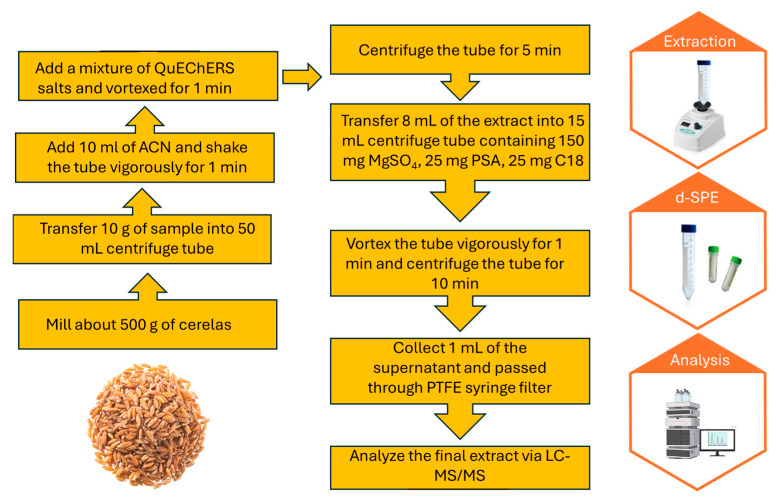
The flowchart of the QuEChERS method procedure used for the cereal sample.

Initially, the grain samples were milled. Subsequently, 10 g of the sample was accurately weighed and transferred into a 50 mL Falcon tube. Then, 10 mL acetonitrile was added to the Falcon tube and vortexed vigorously for 1 min. A mixture of QuEChERS salts, including 1 g of NaCl, 4 g of anhydrous MgSO_4_, and 1.5 g of CH_3_COONa, was added to the solution and vortexed for 1 min. Afterward, the mixture was centrifuged in a Rotina 420R centrifuge for 5 min, and 8 mL of the supernatant solvent was transferred to a 15 mL Falcon tube. Then, 150 mg of anhydrous MgSO_4_, 25 mg of PSA, and 25 mg of C18 were added, and the mixture was vortexed for 1 min. Subsequently, the Falcon tube was centrifuged in a Rotina 420R centrifuge for 10 min. Then, 1 mL of the resulting supernatant was collected, filtered through a 0.45 μm PTFE syringe filter, and injected into the chromatography system. The QuEChERS method is summarized in Figure 3.

### 4.5. Instrumentation Conditions

For the chromatographic analysis, a LC–MS/MS system (Eksigent Ultra LC-100, Eksigent Technologies, Dublin, OH, USA) coupled with tandem mass spectrometry (MS/MS 6500 QTRAP, AB Sciex Instruments, Foster City, CA, USA) was used (Table 4). The chromatographic separations were carried out using a KINETEX C18 column (2.6 μm, 2.1 × 100 mm Phenomenex, Torrance, CA, USA) according to previous protocols [54,55]. Pesticides were detected in multiple reaction monitoring modes (MRM). For each pesticide, the precursor ion and 2 product ions were analyzed. The details of MRM transitions are included in Table 4.

### 4.6. Method Validation

The developed procedure was subject to a validation process according to the guidelines of the European Commission, included in SANTE/11312/2021 “Analytical Quality Control and Method Validation Procedures for Pesticide Residues Analysis in Food and Feed” [56].

### 4.7. Data Analysis

PF was calculated to assess the changes in pesticide residues in cereals during processing using Equation (1):

PF = Cpc/Crac(1)

In the equation, Cpc represents the concentration of pesticide residues in the processed cereal (after ozonation), and Crac represents the concentration in the raw agricultural commodity (unprocessed grain).

A PF value greater than 1 (PF > 1) indicates an increase in pesticide residue levels following processing, while a PF value less than 1 (PF < 1) indicates a reduction in pesticide residues [57,58].

### 4.8. Statistical Analysis

Statistical significance was determined using one-way ANOVA followed by Fisher’s exact test (*p* ≤ 0.05) to analyze differences in pesticide concentrations at specific time points (30 and 60 min). The same letter indicates no significant difference (*p* ≥ 0.05). Statistical analyses were performed using Statistica 12 software (StatSoft, Tulsa, OK, USA).

## 5. Conclusions

It was observed that ozone significantly decreased the content of 12 pesticides in grains of three cereal types (barley, wheat, and rye). All the compounds under analysis were subject to degradation; however, the degradation rate differed between the pesticide and cereal types. In the case of systemic pesticides, the reduction in thiophanate-methyl residues was only 39% in barley grains, whereas the largest drops were recorded for imidacloprid in rye grains—82%. Non-systemic pesticides demonstrated higher and more consistent levels of reduction, falling within the range of 74–94%. In contrast, the lowest value was obtained for beta-cyfluthrin in barley grains, while the highest value was observed for deltamethrin in rye grains. The final effect was the calculation of 72 processing factors for the cereal/process/pesticide combination. The calculated processing factors could be used to supplement European databases of pesticide processing coefficients in cereals.

## Figures and Tables

**Table 1:** Validation parameters of the analytical method for determining 12 systemic and non-systemic pesticides in samples of three cereal grain types (n = 3).

Compound		Wheat			Rye			Barley		
		R, % (RSD, %)	ME, %	U, %	R, % (RSD, %)	ME, %	U, %	R, % (RSD, %)	ME, %	U, %
systemic										
Difenoconazole	0.001	113 (4)	−2	13	98 (8)	12	4	86 (12)	7	6
	0.010	86 (5)	−5	12	78 (8)	−11	9	75 (7)	−7	7
	0.100	73 (7)	8	7	100 (7)	−5	8	94 (2)	8	6
Prochloraz	0.001	75 (2)	5	8	81 (3)	5	9	88 (5)	16	10
	0.010	85 (7)	4	11	83 (8)	−2	10	92 (4)	−8	11
	0.100	73 (6)	4	16	78 (5)	10	17	70 (12)	15	10
Azoxystrobin	0.001	105 (6)	2	10	86 (9)	5	10	88 (2)	−10	12
	0.010	98 (8)	5	15	96 (5)	7	12	89 (7)	9	14
	0.100	78 (5)	8	8	105 (2)	10	6	78 (12)	4	10
Tetraconazole	0.001	83 (7)	8	11	82 (4)	7	7	78 (10)	10	8
	0.010	82 (7)	10	12	86 (5)	12	18	78 (8)	13	14
	0.100	112 (4)	4	7	105 (7)	−8	13	110 (15)	−7	9
Tebuconazole	0.001	74 (5)	6	5	115 (6)	8	12	77 (10)	−5	8
	0.010	78 (7)	8	4	90 (7)	6	8	85 (5)	7	6
	0.100	91 (3)	8	4	94 (4)	7	9	98 (6)	−2	9
Thiophanate methyl	0.001	78 (4)	15	11	85 (5)	−5	11	100 (3)	8	8
	0.010	71 (8)	−6	13	83 (7)	−7	17	76 (6)	−10	10
	0.100	98 (2)	5	10	92 (12)	8	6	99 (4)	−9	12
Imidacloprid	0.001	115 (2)	−3	14	95 (5)	11	5	118 (12)	6	8
	0.010	87 (5)	−7	10	88 (8)	−12	9	75 (7)	−7	9
	0.100	75 (4)	2	8	104 (3)	−4	7	99 (3)	8	6
non-systemic										
Deltamethrin	0.001	78 (8)	4	8	116 (6)	8	8	88 (10)	−5	8
	0.010	88 (7)	6	9	92 (7)	4	11	86 (9)	10	8
	0.100	92 (5)	10	6	102 (4)	10	9	98 (6)	−6	10
Cypermethrin	0.001	89 (7)	10	12	82 (5)	−8	11	105 (4)	19	6
	0.010	45 (8)	−3	10	78 (7)	−3	12	116 (6)	−10	8
	0.100	96 (7)	2	8	86 (12)	5	6	97 (2)	−9	5
beta-Cyfluthrin	0.001	77 (4)	5	11	85 (5)	−5	11	100 (3)	8	8
	0.010	71 (8)	−6	13	83 (7)	−7	17	76 (6)	−10	10
	0.100	98 (2)	5	10	92 (12)	8	6	99 (4)	−9	12
Pirimiphos-methyl	0.001	100 (7)	6	14	112 (6)	10	12	104 (8)	12	14
	0.010	76 (11)	11	16	79 (10)	9	14	71 (13)	17	18
	0.100	73 (5)	9	10	74 (5)	4	10	85 (13)	8	5
Prosulfocarb	0.001	109 (5)	−5	12	99 (5)	8	3	88 (10)	−5	6
	0.010	75 (2)	−7	12	79 (9)	−11	8	75 (7)	6	8
	0.100	72 (4)	8	5	102 (5)	−4	7	94 (2)	9	13

Note: R: recovery; RSD: relative standard deviation; ME: matrix effect; U: uncertainty.

**Table 2:** Effect of ozonation on the concentration of pesticide residues in cereals.

Pesticide	Group	Ozone Treatment	Concentration (mg kg^−1^)			Means ± SD (mg kg^−1^)
			Raw	30 min	60 min	
systemic						
Difenoconazole	I	barley	0.7148	0.2977	0.2632	0.2805 ± 0.024 ^bc^
		wheat	0.5252	0.3484	0.2451	0.2968 ± 0.073 ^b^
		rye	0.4256	0.2022	0.1662	0.1842 ± 0.025 ^g^
Prochloraz	I	barley	0.5492	0.2532	0.1235	0.1884 ± 0.092 ^g^
		wheat	0.5776	0.2344	0.1164	0.1754 ± 0.083 ^g^
		rye	0.5792	0.1962	0.0841	0.1402 ± 0.079 ^h^
Azoxystrobin	II	barley	0.3224	0.2576	0.1651	0.2114 ± 0.065 ^e^
		wheat	0.4480	0.3199	0.2264	0.2732 ± 0.066 ^c^
		rye	0.4320	0.2935	0.1741	0.2338 ± 0.084 ^d^
Tetraconazole	III	barley	0.4412	0.3662	0.2465	0.3064 ± 0.085 ^b^
		wheat	0.5278	0.3232	0.2502	0.2867 ± 0.052 ^bc^
		rye	0.4032	0.2221	0.1673	0.1947 ± 0.039 ^fg^
Tebuconazole	III	barley	0.4012	0.3165	0.1765	0.2465 ± 0.099 ^d^
		wheat	0.4776	0.3183	0.1621	0.2402 ± 0.110 ^d^
		rye	0.3692	0.2588	0.1115	0.1852 ± 0.104 ^g^
Thiophanate methyl	IV	barley	0.6216	0.4519	0.3812	0.4166 ± 0.050 ^a^
		wheat	0.4608	0.2851	0.2656	0.2754 ± 0.014 ^c^
		rye	0.4212	0.2515	0.1971	0.2243 ± 0.038 ^e^
Imidacloprid	V	barley	0.1996	0.1178	0.0821	0.1000 ± 0.025 ^j^
		wheat	0.1888	0.1256	0.0966	0.1111 ± 0.021 ^i^
		rye	0.2044	0.1365	0.0378	0.0872 ± 0.070 ^k^
non-systemic						
Deltamethrin	VI	barley	0.1864	0.0668	0.0281	0.0475 ± 0.027 ^m^
		wheat	0.3072	0.0807	0.405	0.2429 ± 0.229 ^d^
		rye	0.4236	0.0955	0.0232	0.0594 ± 0.051 ^m^
Cypermethrin	VI	barley	0.2888	0.1066	0.0644	0.0855 ± 0.030 ^kl^
		wheat	0.9656	0.4362	0.1261	0.2812 ± 0.219 ^bc^
		rye	0.8312	0.2826	0.1252	0.2039 ± 0.111 ^e^
beta-Cyfluthrin	VI	barley	0.2928	0.1523	0.0821	0.1172 ± 0.050 ^i^
		wheat	0.6407	0.2615	0.1561	0.2088 ± 0.075 ^ef^
		rye	0.6836	0.2782	0.1577	0.2180 ± 0.085 ^e^
Pirimiphos-methyl	VII	barley	0.3708	0.1255	0.0723	0.0989 ± 0.038 ^k^
		wheat	0.3624	0.1038	0.0556	0.0797 ± 0.034 ^l^
		rye	0.3258	0.0865	0.0186	0.0526 ± 0.048 ^m^
Prosulfocarb	VIII	barley	0.2464	0.1531	0.0931	0.1231 ± 0.042 ^i^
		wheat	0.3318	0.2044	0.1141	0.1593 ± 0.064 ^h^
		rye	0.2488	0.1228	0.0782	0.1005 ± 0.032 ^j^

Chemical group: I—Conazole; II—Strobilurin; III—Triazole; IV—Carbamate; V—Neonicotinoid; VI—Pyrethroid; VII—Organophosphate; VIII—Thiocarbamate; Raw: unprocessed samples; 30 min: samples processed by ozonation for 30 min; 60 min: samples processed by ozonation for 60 min. The letters of statistical significance refer to the differences in pesticide concentration between cereals. The same letter indicates statistical significance (*p* < 0.05).

**Table 4:** LC–MS/MS conditions for analyzing 12 pesticides.

Instrument	Eksigent Ultra LC-100 with MS/MS 6500 QTRAP			
Column	KINETEX C18 column (2.6 μm, 2.1 × 100 mm)			
Mobile phase	A: water with 0.2% formic acid and 5 mM ammonium formate B: methanol with 0.2% formic acid and 5 mM ammonium formate			
Gradient table	Time (min)	A (%)		B (%)
	0.0	99		1
	0.5	99		1
	5.0	10		90
	7.0	10		90
	8.0	99		1
	10.0	99		1
Flow rate	0.5 mL min^−1^			
Column temp.	40 °C			
Injection volume	10 μL			
MRM condition	MRM Pair	MRM 1 (quantitative)	MRM 2 (qualitative)	Retention time (min)
	Difenoconazole	406 > 251	406 > 188	5.80
	Prochloraz	376 > 307.9	376 > 70	5.40
	Azoxystrobin	404.1 > 371.9	404.1 > 344	5.05
	Tetraconazole	372 > 159	372 > 70	5.35
	Tebuconazole	308.1 > 70	308.1 > 125.1	5.55
	Thiophanate methyl	343 > 151	343 > 192	4.20
	Imidacloprid	256 > 209.1	256 > 175.1	3.20
	Deltamethrin *	525 > 507.8	522.9 > 280.8	6.80; 6.90
	Cypermethrin *	433.2 > 191	433.2 > 416.1	6.70; 6.75; 6.90
	beta-Cyfluthrin *	451 > 206	451 > 191	6,65
	Pirimiphos-methyl	306.9 > 164.1	306.9 > 108	5.05; 5.50
	Prosulfocarb	252.1 > 91; 252.1	252.1 > 128.1	5.95
Ionization mode	ESI Positive			

* reported as the sum of isomers.

## Data Availability

The original contributions presented in this study are included in the article. Further inquiries can be directed to the corresponding author.

## Abbreviations

The following abbreviations are used in this manuscript:

R	Recovery
RSD	Relative standard deviation
ME	Matrix effect
U	Uncertainty
PF	Processing factor
TPP	Triphenyl phosphate
ISs	Internal standards
d-SPE	Dispersive solid-phase extraction
MgSO_4_	Magnesium Sulfate
PSA	Primary Secondary Amine
C18	Octadecylsilane
M	Molar mass
P	Logarithm of the octanol-water partition coefficient
MRM	Multiple Reaction Monitoring
Cpc	Concentration of pesticide residues in the processed cereal
Crac	Concentration in the raw agricultural commodity
